# Interplay Between Viral Shedding, Age, and Symptoms in Individual Infectivity of COVID-19 Breakthrough Infections in Households

**DOI:** 10.3390/vaccines13030329

**Published:** 2025-03-19

**Authors:** Shuaibing Dong, Ying Sun, Shuyu Ni, Yi Tian, Zhaomin Feng, Lei Jia, Xiaoli Wang, Daitao Zhang, Quanyi Wang, Tim K. Tsang, Peng Yang

**Affiliations:** 1 Beijing Key Laboratory of Surveillance, Early Warning and Pathogen Research on Emerging Infectious Diseases, Beijing Center for Disease Prevention and Control, Beijing 100013, China; dongshuaibing158@126.com (S.D.); sun1ying2@163.com (Y.S.); shuyu_ni98@163.com (S.N.); tianyixingfu_1@163.com (Y.T.); fengzhaomin214@163.com (Z.F.); lailajia@126.com (L.J.); wangxiaoli198215@163.com (X.W.); zdt016@163.com (D.Z.); bjcdcxm@126.com (Q.W.); 2 Beijing Research Center for Respiratory Infectious Diseases, Beijing 100013, China; 3 School of Public Health, Capital Medical University, Beijing 100069, China; 4 WHO Collaborating Centre for Infectious Disease Epidemiology and Control, School of Public Health, Li Ka Shing Faculty of Medicine, The University of Hong Kong, Hong Kong, China; 5 Laboratory of Data Discovery for Health, Ltd., Hong Kong Science and Technology Park, New Territories, Hong Kong, China

**Keywords:** COVID-19, breakthrough infections, household transmission, viral load, symptoms

## Abstract

Background/Objectives: Understanding the factors influencing breakthrough infections following COVID-19 vaccination is critical for disease prevention, especially in households where transmission risks are high. Factors such as age, symptoms, living conditions, and viral load contribute to household transmission dynamics. Methods: To elucidate this complex interplay of these factors, we analyzed a detailed household transmission study of COVID-19 involving 839 households and 1598 vaccinated individuals during the Omicron variant outbreak in Beijing, China, from April to June 2022. Using multivariate logistic regression models, we analyzed the impact of demographic, environmental, clinical, and virological factors on the risk of breakthrough infections. Results: In multivariate analysis. we estimated that index cases aged 45–59 and 60+ years were associated with 80% (95% confidence interval [CI]: 35%, 140%) and 288% (95% CI: 160%, 481%) higher infectivity compared with index cases aged 18–44 years. We estimated that index cases with fever, headache and cough were associated with 43% (95% CI: 11%, 84%), 78% (95% CI: 18%, 168%) and 67% (25%, 123%) higher infectivity compared with those without. Index cases with higher viral loads were associated with higher infectivity in univariate analysis, but this was no longer significant in multivariate analysis. Smaller living space and two-member households were associated with higher odds of breakthrough infections. Conclusions: Age, symptoms, and living conditions were significant risk factors for breakthrough infections during the Omicron outbreak. Suburban settings, smaller spaces, and two-member households enhance transmission risks. These findings inform targeted interventions to reduce household transmission.

## 1. Introduction

COVID-19, caused by severe acute respiratory syndrome coronavirus-2 (SARS-CoV-2), has had a profound impact on global health and economic development [1,2]. Vaccination against SARS-CoV-2 offers high protection against severe infection and mortality, serving as the cornerstone strategy for mitigating the COVID-19 pandemic worldwide [3,4]. However, even with widespread vaccination, there remains a risk of infection and transmission, particularly during the Omicron-dominant period, highlighting the need for studies on vaccine breakthrough infections to inform public health policies and prevent future outbreaks [5,6,7].

The emergence of breakthrough infections is influenced by various factors. Current research has primarily focused on SARS-CoV-2 variants, vaccine effectiveness over time, and demographic characteristics of affected populations [8,9]. Variants of concern (VOCs) have been identified as a major driver of breakthrough infections [8]. Regardless of the vaccine type, breakthrough infections can occur, with risk factors commonly including older age, male sex, high viral load, poor lifestyle habits (e.g., smoking and alcohol consumption), employment in healthcare, and underlying conditions, such as respiratory, cardiovascular, and immune system diseases [9,10]. Additionally, vaccine type may influence breakthrough risk, with studies showing that Ad26.COV2.S and ChAdOx1 are associated with a higher risk compared to BNT162b2, while mRNA-1273 appears to confer a lower risk [11].

Despite these insights, there is a notable gap in research on breakthrough infections associated with inactivated vaccines. Moreover, many previous studies failed to account for critical confounders, such as the exposure history of close contacts or variations in transmissibility across different groups. In contrast, our study incorporates these factors to provide a more comprehensive analysis. Furthermore, no existing research has systematically examined the risk factors for breakthrough infections based on the characteristics, vaccination status, and household living conditions of index and secondary cases.

To address these gaps, this study employs a robust retrospective cohort design centered on families to systematically analyze the risk factors for breakthrough infections during the Omicron variant epidemic. The household setting is particularly suitable for such research, as COVID-19 transmission risk is approximately four times higher in households than in other settings [12]. This setting also ensures reliable information on transmissions, as household contacts are more likely to be exposed due to their close proximity to index cases. The findings aim to provide valuable guidance for the future prevention and control of COVID-19 outbreaks.

## 2. Materials and Methods

### 2.1. Data Source

The data on COVID-19 cases and their close contacts are sourced from the National Notifiable Disease Report System and on-site epidemiological investigations. In this system, cases were identified through both mass testing campaigns and from individuals with symptoms who sought medical care. The collected data included information on age, gender, vaccination status, exposure status, residential areas, living arrangements, residential floor level, living space, number of co-residents, bathroom conditions, mask-wearing habits, case cycle threshold (Ct) values, and case symptoms.

### 2.2. Definitions

Close contacts were identified as those who had been in close contact with individuals infected with SARS-CoV-2 within the four days preceding the onset of symptoms or, in the case of asymptomatic infections, within four days prior to sampling, and who had not effectively protected themselves. Our study focused on close contacts with high household exposure risk who had received at least two doses of inactivated COVID-19 vaccines to investigate breakthrough infections. Households without index cases were excluded to eliminate the potential influence of common exposures outside the household.

Breakthrough infections were defined as positive COVID-19 test results through RT–PCR, occurring more than 14 days after completing the recommended vaccination regimen [13].

Unvaccinated comprises those individuals who had no history of COVID-19 vaccination before viral exposure. Primary encompasses those who had received 2 doses of the inactivated vaccine >14 days before exposure. Booster comprises individuals who have completed a 3-dose regimen of the inactivated COVID-19 vaccine, with the final dose administered >7 days before exposure [14].

### 2.3. Statistical Analysis

The secondary attack rate is defined as the proportion of breakthrough infections among household contacts. Differences in attack rates across subgroups of close contacts were compared using Chi-squared tests, with statistical significance defined as *p* < 0.05. Univariate and multivariate logistic regression models were employed to identify potential risk factors for breakthrough infections following COVID-19 vaccination.

Our modeling strategy comprised a sequential logistic regression analysis. We began with univariate logistic regressions to identify predictors significantly associated with breakthrough infections. All the variables were then put into a stepwise model selection and these provided the minimum Akaike Information Criteria (AIC) included in our multivariate analysis. Basic factors (age and sex) and vaccination status were also adjusted in our final multivariate logistic regression model. In a subsequent step, we added index case characteristics such as symptom profiles and viral load measurements, to the model to determine their additional impact on infection risk. This systematic, stepwise approach allowed us to isolate the independent effects of each variable on the risk of breakthrough infections. Various demographic, environmental, and clinical variables associated with the secondary attack rate among household close contacts were explored.

Demographic factors included the age group (e.g., 18–44, 45–59) and sex (male vs. female) of both household contacts and index cases. Vaccination status was assessed for both groups, including primary series, booster doses, and unvaccinated individuals, as well as the interval between the last dose and exposure (e.g., 1–3, 3–6 months).

Environmental factors included the area of residence (urban vs. suburban), whether the household was a single-story house, the living floor (e.g., 1–3, 4–9, or underground), the size of the living space (<100 vs. ≥100 square meters), the number of co-residents (e.g., >5, 3–5, or 2), and the presence of separate bathrooms. To assess the potential impact of environmental conditions on transmission risk, we collected information on housing type and residential floor to capture differences in ventilation and crowding. While some studies have found that higher residential floors may correlate with higher socioeconomic status and better living conditions, our study in Beijing did not include direct socioeconomic measures. Thus, our analysis focused solely on the environmental aspects, such as ventilation and the potential for crowding, that are directly associated with transmission risk. Previous studies have shown that reduced ventilation in underground or lower-floor residences can increase the risk of respiratory infections [15,16].

Clinical variables related to index cases included their habit of wearing masks, their cycle threshold (Ct) value (e.g., >30, 25–30, <25), and the presence of specific symptoms, such as fever, headache, sore throat, and persistent cough. In the multivariate analyses, all factors associated with the odds of breakthrough infections were included. Meanwhile, in our analyses, the reference category was selected based on clinical relevance and the observed frequency distribution, so that we designated the 18–44 age group as the reference category. This choice reflects both the frequency distribution observed in our sample and epidemiological evidence indicating that individuals in this age bracket generally have a lower risk of severe outcomes from COVID-19. This clinical relevance provides a solid baseline for comparing risk estimates across older age groups. Data cleaning was conducted using Microsoft Excel 2010 (Microsoft Office, Los Angeles, CA, USA), and statistical analyses were performed using R version 4.2.2 (https://www.R-project.org/; The R Foundation, Vienna, Austria).

## 3. Results

### 3.1. Demographic Characteristics of the Household Index Case and Households Close Contacts

From 1 April to 30 June 2022, a total of 839 index cases and 1598 vaccinated household close contacts were included in the analysis. Among the 839 index case-patients, 428 (51.01%) were aged 18–44 years, 361 (43.03%) were male, and 586 (69.85%) resided in urban areas. Among the 1598 vaccinated household close contacts, 56.01% were male, and 54.76% were aged 18–44 years (Table 1).

**Figure 1:**
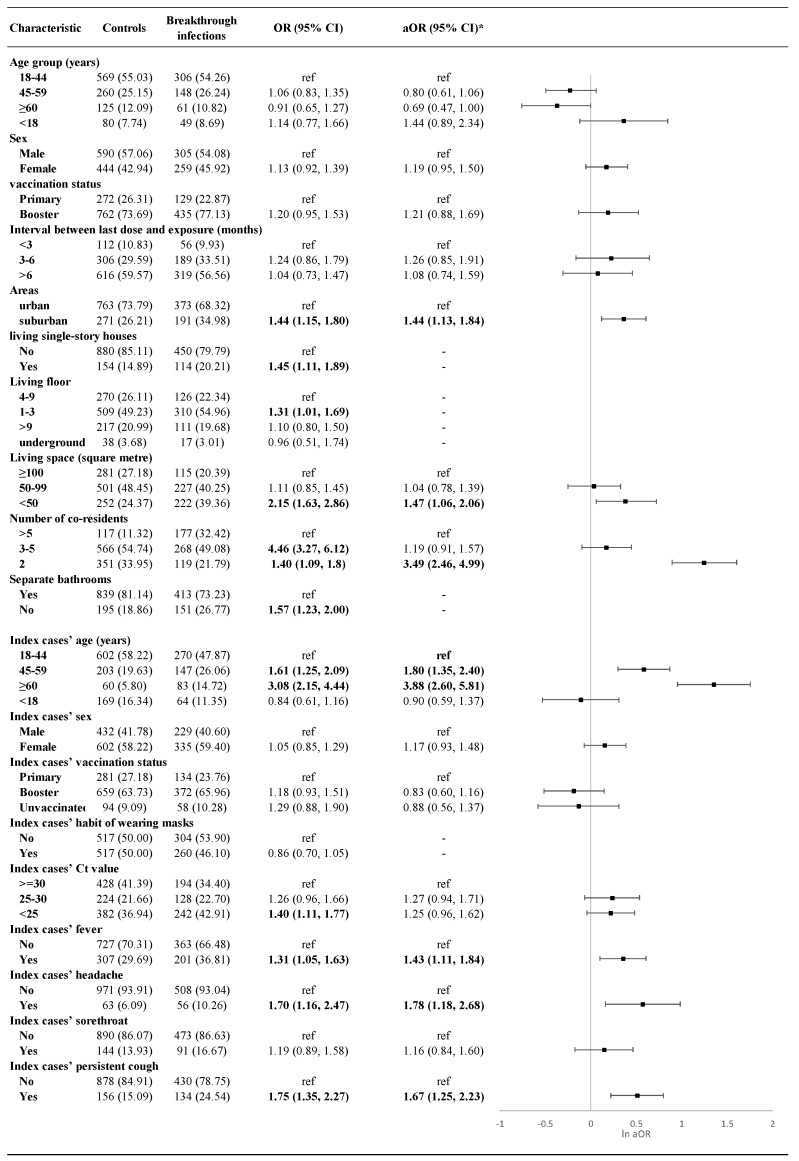
Demographic characteristics, living conditions and index case information for participants and risk factors analysis breakthrough infections. *: adjusted for age, sex, vaccination status, interval between last dose and exposure, areas, living space, number or co-residents, and index cases’ age, sex, vaccination status, habit of wearing masks, Ct value and symptoms.

Nearly all participants received inactivated COVID-19 vaccines; specifically, over 99% of the study population was immunized with BBIBP-CorV (manufactured by Sinopharm Group, Beijing, China) and CoronaVac (manufactured by Sinovac Life Sciences Co., Ltd, Beijing, China). Of these, 1197 (74.91%) had received a booster dose; however, 935 (58.51%) had an interval exceeding six months between their last dose and exposure. Additionally, 1136 (71.09%) resided in urban areas, 819 (51.25%) lived on the first to third floors of buildings, and 661 (41.36%) lived in households with 3–5 co-residents (Figure 1).

### 3.2. Attack Rate Among Close Contacts

Among the 1598 household close contacts, 564 individuals experienced breakthrough infections, resulting in an overall secondary attack rate of 35.29% (564 of 1598) following full vaccination. The attack rate was significantly higher among individuals living in urban areas compared to those in suburban areas (*p* = 0.002). Households with only two co-residents exhibited a higher attack rate compared to households with more than two co-residents (*p* < 0.001), and households without separate bathrooms had a higher attack rate compared to those with separate bathrooms (*p* < 0.001).

The attack rate was also higher among individuals living in single-story houses (*p* = 0.008), whereas larger living areas were associated with lower attack rates. Significant differences in attack rates were observed based on the age of the household index case (*p* < 0.001), with older index cases associated with higher attack rates. Furthermore, the occurrence of headache and persistent cough in the primary case was significantly associated with higher attack rates among household close contacts (*p* < 0.001) (Table 2).

### 3.3. Risk Factors of Breakthrough Infections

Univariate analyses revealed that living in suburban areas, residing in single-story houses, living on the first to third floors, having a living area of less than 50 square meters, living with fewer than five co-residents, and residing in households without a separate bathroom were significant risk factors for breakthrough infections. Additionally, the presence of an elderly household index case, a higher viral load (Ct < 25), and symptoms such as fever, headache, and persistent cough in the index case were associated with an increased likelihood of breakthrough infections among household close contacts.

After adjusting for sex and other risk factors identified in the univariate analyses, the results showed that vaccinated close contacts residing in suburban areas had a higher risk of breakthrough infections compared to those in urban areas (OR = 1.44, 95% CI: 1.13–1.84). Vaccinated close contacts with a living space of less than 50 square meters had 1.47 times the odds of breakthrough infection compared to those with a living space of ≥100 square meters (OR = 1.47, 95% CI: 1.06–2.06). Household close contacts with only two co-residents had significantly higher odds of breakthrough infections compared to those with more than five co-residents (OR = 3.49, 95% CI: 2.46–4.99). Furthermore, households with older primary cases had greater odds of breakthrough infections (Reference: 18–44 years; 45–59 years: OR = 1.80 [95% CI: 1.35–2.40]; ≥60 years: OR = 3.88 [95% CI: 2.60–5.81]). Fever (OR = 1.43, 95% CI: 1.11–1.84), headache (OR = 1.78, 95% CI: 1.18–2.68), and persistent cough (OR = 1.67, 95% CI: 1.25–2.23) in the index case were also significantly associated with a higher risk of breakthrough infections among household close contacts (Figure 1).

**Figure 2:**
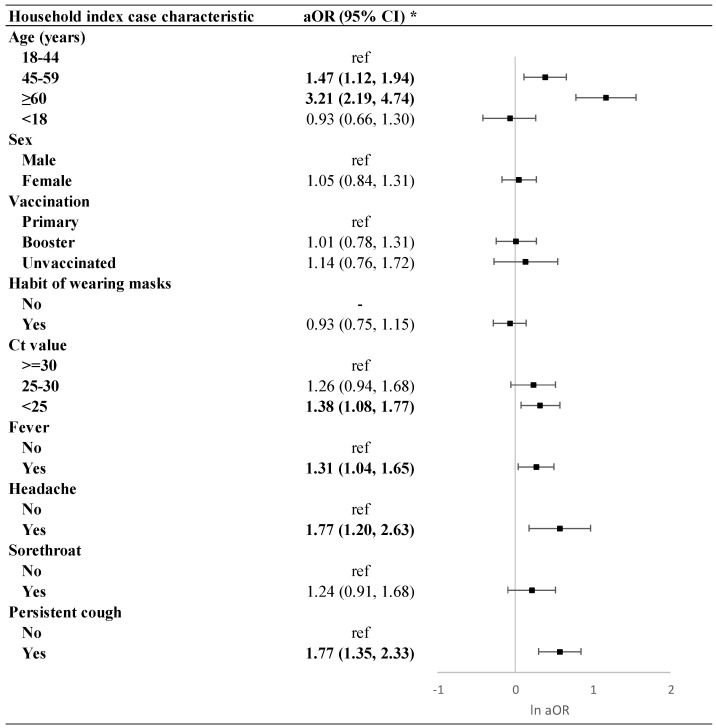
Adjusting for all demographic and household conditions, impact of the index case’s characteristics on breakthrough infections among household close contacts. *: adjusting for all demographic and household conditions.

Additionally, we conducted an analysis in which the characteristics of the index case were added sequentially, adjusting for demographic and household condition variables. This approach allowed us to evaluate the impact of the index case’s characteristics on breakthrough infections among household close contacts. The analysis found that the index case’s age, Ct value, and symptoms (excluding sore throat) were associated with higher odds of infection in close contacts. Compared to index cases with a Ct > 30, those with a Ct < 25 significantly increased the risk of breakthrough infections (OR = 1.38, 95% CI: 1.08–1.77) (Figure 2). However, when the index case’s age and symptoms were considered, the Ct value was no longer associated with higher odds of infection. Conversely, the age and symptoms of the index case remained significantly associated with higher odds of infection among close contacts.

## 4. Discussion

In our research, we examined the factors associated with COVID-19 breakthrough infections after vaccination, using a real-world retrospective household transmission study based on detailed contact tracing data from Beijing. Cohabitating family members of COVID-19 patients were at higher risk of infection due to the inevitable high-frequency of prolonged unprotected exposure [17,18]. Furthermore, the demographic information for both infectors and infectees was recorded, making the data more reliable than in retrospective cohort studies or observational studies focused on close contacts in other settings, such as workplaces. This detailed dataset allowed us to disentangle how age, vaccination status, symptoms, virological factors (Ct value), and environmental conditions collectively influenced the infectivity and susceptibility of breakthrough infections.

First, age played a critical role in infectivity after adjusting vaccination status, Ct value and symptom of cases. We observed that vaccinated close contacts in households with older primary cases had significantly greater odds of acquiring breakthrough infections. A study with similar design, carried out in India during the spread of the B.1.617.2 Delta variant, reported that the age of the index case (OR = 1.03, 95% CI: 1.01–1.04) was associated with secondary infections [19]. Likewise, a cohort study conducted in Spain during the dominance of the B.1.177 variant found that the risk of transmission increased with the age of index cases [20]. However, our study did not find a significant relationship between the age of close contacts and breakthrough infections, contrasting with earlier findings of increased susceptibility with age for earlier variants, like Delta. This shift aligns with observations in other studies [21,22,23,24]. which suggest that the higher transmissibility of Omicron, combined with greater social interactions among younger populations in settings such as schools, may have increased exposure while reducing vaccine effectiveness against infection compared to earlier variants, like Delta [25].

After adjusting for index cases’ age and Ct value, we found that the onset of fever, headache, and persistent cough symptoms in the primary case was associated with a higher risk of breakthrough infection among household close contacts. Similar findings have been reported in multiple studies. For example, during the B.1.617.2 Delta variant wave in India, symptoms in index cases, particularly cough, were significantly associated with secondary infections [10,19]. Likewise, a study conducted in Spain during the dominance of the B.1.177 variant found that the risk of transmission was higher from symptomatic index cases [20]. Furthermore, a study in Australia reported that symptomatic primary cases had higher odds of secondary transmission compared to asymptomatic cases [26]. A potential explanation for these findings is that symptomatic cases are often more severe, requiring closer care from household members, which increases both the intensity and duration of exposure. This increased exposure may amplify the likelihood of breakthrough infections among close contacts [27].

In the univariate analysis, we found that a higher viral load in the primary case was associated with an increased risk of COVID-19 transmission within households. However, this association was not statistically significant in the multivariate analysis. A study conducted in the United States similarly found that, after adjusting for factors such as cough, time between testing and exposure, and physical contact, a higher viral load in the primary case was significantly associated with an increased risk of transmission to close contacts [10]. Another study predicted that higher viral load levels could increase the probability of transmission to household contacts by 24% to 58% [28]. Several explanations could account for these findings. One potential reason is the insufficient sample size for detection of a significant association between viral shedding and infectivity after adjusting for multiple factors, including age and symptoms, as viral loads are generally expected to correlate with infectiousness [10]. Another possibility is that viral loads measured in the throat or nose might not precisely reflect the quantity of infectious viruses present in exhaled breath. Previous research on influenza, for example, reported a low correlation between viral loads in respiratory samples and infectiousness via exhaled breath [16]. Additionally, in densely populated household environments with frequent and prolonged contact, even patients with low viral loads may effectively transmit the virus, making the exact viral load less critical in these settings [15]. Our findings suggest that, while high viral loads may serve as an indicator of higher infectiousness when other information is unavailable, they lose their importance in determining infectivity when factors such as age and fever are considered. This implies that precise viral load measurements may not be an absolute necessity for identifying individuals with higher infectiousness, similar to the case of influenza [29].

We also investigated the environmental factors associated with breakthrough infections and found that vaccinated close contacts residing in suburban areas had a higher risk of breakthrough infection compared to those living in urban areas. Vaccinated close contacts with a living space of less than 50 m^2^ had 1.47 times the odds of breakthrough infection compared to those with a living space of 100 m^2^ or more. Additionally, our study indicated that households with only two members exhibited a higher risk of secondary transmission, likely due to the closer contact between spouses. Previous studies have also identified spousal relationships with the index case as a significant risk factor for SARS-CoV-2 transmission within households [26,30,31]. These findings underscore the importance of targeted public health measures for specific groups, such as non-urban residents, individuals living in small spaces, and those in spousal relationships. Despite being fully vaccinated, these individuals should continue adhering to preventive measures, including wearing masks, frequent handwashing, and maintaining social distancing, to mitigate the risk of breakthrough infections.

As highlighted by previous studies, the type of vaccine can influence the likelihood of breakthrough infections. It is important to note that our cohort was exclusively vaccinated with inactivated vaccines—BBIBP-CorV and CoronaVac—which, due to their similar distribution in our sample, precluded a statistically robust comparison of vaccine-specific breakthrough risks. This limitation is acknowledged in the discussion of vaccine effectiveness differences observed in studies of mRNA vaccines. A prospective cohort study in Belgium indicated that individuals vaccinated with Ad26.COV2.S or ChAdOx1 faced a higher likelihood of breakthrough infection compared to BNT162b2, while mRNA-1273 vaccination was associated with a lower risk [9]. Similarly, a study conducted in Brazil found that, compared to individuals vaccinated with the Vaxzevria vaccine, those vaccinated with CoronaVac faced a higher risk of unfavorable outcomes, whereas those vaccinated with Comirnaty had a significantly lower risk [6]. Additionally, Liu et al. found that individuals vaccinated with Pfizer/BNT162b2 were at the highest risk for breakthrough infections, with an incidence rate ratio of 1.66 (95% CI: 1.17–2.35), when compared to those vaccinated with Moderna/mRNA-1273 [32].

This study has several limitations. First, as a retrospective observational study, it cannot rule out the influence of unidentified confounders. Second, the study was conducted during the Omicron BA.2 variant outbreak, with nearly all participants receiving inactivated COVID-19 vaccines, limiting the generalizability to other vaccine types and variant strains. Third, the likelihood of testing was higher among certain vulnerable populations, such as the elderly, which may have introduced bias into the findings. Finally, we did not account for the potential of tertiary transmission and infection acquired from outside households, as we assumed that all secondary cases were infected by index cases, a limitation which could be overcome by more advanced household transmission models.

## 5. Conclusions

This study identifies key factors influencing COVID-19 breakthrough infections in household settings, including older age and symptoms such as fever, headache, and persistent cough in primary cases, as well as environmental factors, like smaller living spaces, suburban residence, and spousal relationships. Viral load was initially associated with increased risk, but its significance diminished after adjusting for age and symptoms. These findings highlight the need for continued public health measures and targeted strategies, including booster vaccinations and adherence to preventive practices, to reduce household transmission.

## Figures and Tables

**Table 1:** Characteristics of 839 household index case-patients and 1598 vaccinated close contacts households with COVID-19 outbreaks, Beijing, China.

Characteristic	Household Index Case *n* (%)	Close Contacts Household *n* (%)
Age group (years)		
18–44	428 (51.01)	875 (54.76)
45–59	212 (25.27)	408 (25.53)
≥60	95 (11.32)	186 (11.64)
<18	104 (12.40)	129 (8.07)
Sex		
Male	361 (43.03)	895 (56.01)
Female	478 (56.97)	703 (43.99)
vaccination status		
Primary	202 (24.08)	401 (25.09)
Booster	551 (65.67)	1197 (74.91)
Unvaccinated	86 (10.25)	0 (0.00)
Interval between last dose and exposure (months)		
<3	80 (9.54)	168 (10.51)
3–6	171 (20.38)	495 (30.98)
>6	588 (70.08)	935 (58.51)
Areas		
urban	586 (69.85)	1136 (71.09)
suburban	253 (30.15)	462 (28.91)
living in single-story houses		
No	676 (80.57)	1330 (83.23)
Yes	163 (19.43)	268 (16.77)
Living floor		
4–9	208 (24.79)	396 (24.78)
1–3	426 (50.77)	819 (51.25)
>9	165 (19.67)	328 (20.53)
underground	30 (3.58)	55 (3.44)
Living space (square meters)		
≥100	189 (22.53)	396 (24.78)
50–99	358 (42.67)	728 (45.56)
<50	292 (34.8)	474 (29.66)
Number of co-residents		
>5	149 (17.76)	524 (32.79)
3–5	456 (54.35)	661 (41.36)
2	234 (27.89)	413 (25.84)
Separate bathrooms		
Yes	639 (76.16)	1252 (78.35)
No	200 (23.84)	346 (21.65)

**Table 2:** Attack rate of household close contacts, Beijing, China.

Characteristic	Attack Rate, %	χ^2^	*p* Value
Age group (years)		1.129	0.77
18–44	34.97 (306/875)		
45–59	36.27 (148/408)		
≥60	32.80 (61/186)		
<18	37.98 (49/129)		
Sex		1.199	0.274
Male	34.08 (305/895)		
Female	36.84 (259/703)		
vaccination		2.110	0.146
Primary	32.17 (129/401)		
Booster	36.34 (435/1197)		
Interval between last dose and exposure (months)		2.657	0.265
<3	33.33 (56/168)		
3–6	38.18 (189/495)		
>6	34.12 (319/935)		
Areas		10.040	0.002
urban	32.83 (373/1136)		
suburban	41.34 (191/462)		
living in single-story houses		7.021	0.008
No	33.83 (450/1330)		
Yes	42.54 (114/268)		
Living floor		5.206	0.157
4–9	31.82 (126/396)		
1–3	37.85 (310/819)		
>9	33.84 (111/328)		
underground	30.91 (17/55)		
Living space (square meters)		39.820	<0.001
≥100	29.04 (115/396)		
50–99	31.18 (227/728)		
<50	46.84 (222/474)		
Number of co-residents		104.005	<0.001
>5	25.32 (119/470)		
3–5	32.13 (268/834)		
2	60.20 (177/294)		
Separate bathrooms		13.012	<0.001
Yes	32.99 (413/1252)		
No	43.64 (151/346)		
Index cases’ age (years)		52.704	<0.001
18–44	30.96 (270/872)		
45–59	42.00 (147/350)		
≥60	58.04 (83/143)		
<18	27.47 (64/233)		
Index cases’ sex		0.163	0.687
Male	34.64 (229/661)		
Female	35.75 (335/937)		
Index cases’ vaccination status		2.467	0.291
Primary	32.29 (134/415)		
Booster	36.08 (372/1031)		
Unvaccinated	38.16 (58/152)		
Index cases’ habit of wearing masks		2.069	0.15
No	33.46 (260/777)		
Yes	37.03 (304/821)		
Index cases’ Ct value		7.941	0.687
≥30	31.19 (194/622)		
25–30	36.36 (128/352)		
<25	38.78 (242/624)		
Index cases’ fever		5.683	0.291
No	33.30 (363/1090)		
Yes	39.57 (201/508)		
Index cases’ headache		7.246	0.001
No	34.35 (508/1479)		
Yes	47.06 (56/119)		
Index cases’ sore throat		1.248	0.264
No	34.70 (473/1363)		
Yes	38.72 (91/235)		
Index cases’ persistent cough		17.896	<0.001
No	32.87 (430/1308)		
Yes	46.21 (134/290)		

## Data Availability

The original contributions presented in this study are included in the article.
